# Drug-Selected Human Lung Cancer Stem Cells: Cytokine Network, Tumorigenic and Metastatic Properties

**DOI:** 10.1371/journal.pone.0003077

**Published:** 2008-08-27

**Authors:** Vera Levina, Adele M. Marrangoni, Richard DeMarco, Elieser Gorelik, Anna E. Lokshin

**Affiliations:** 1 University of Pittsburgh Cancer Institute, Pittsburgh, Pennsylvania, United States of America; 2 Department of Medicine, University of Pittsburgh Cancer Institute, Pittsburgh, Pennsylvania, United States of America; 3 Department of Pathology, University of Pittsburgh Cancer Institute, Pittsburgh, Pennsylvania, United States of America; 4 Department of Immunology, University of Pittsburgh Cancer Institute, Pittsburgh, Pennsylvania, United States of America; Dresden University of Technology, Germany

## Abstract

**Background:**

Cancer stem cells (CSCs) are thought to be responsible for tumor regeneration after chemotherapy, although direct confirmation of this remains forthcoming. We therefore investigated whether drug treatment could enrich and maintain CSCs and whether the high tumorogenic and metastatic abilities of CSCs were based on their marked ability to produce growth and angiogenic factors and express their cognate receptors to stimulate tumor cell proliferation and stroma formation.

**Methodology/Findings:**

Treatment of lung tumor cells with doxorubicin, cisplatin, or etoposide resulted in the selection of drug surviving cells (DSCs). These cells expressed CD133, CD117, SSEA-3, TRA1-81, Oct-4, and nuclear β-catenin and lost expression of the differentiation markers cytokeratins 8/18 (CK 8/18). DSCs were able to grow as tumor spheres, maintain self-renewal capacity, and differentiate. Differentiated progenitors lost expression of CD133, gained CK 8/18 and acquired drug sensitivity. In the presence of drugs, differentiation of DSCs was abrogated allowing propagation of cells with CSC-like characteristics. Lung DSCs demonstrated high tumorogenic and metastatic potential following inoculation into SCID mice, which supported their classification as CSCs. Luminex analysis of human and murine cytokines in sonicated lysates of parental- and CSC-derived tumors revealed that CSC-derived tumors contained two- to three-fold higher levels of human angiogenic and growth factors (VEGF, bFGF, IL-6, IL-8, HGF, PDGF-BB, G-CSF, and SCGF-β). CSCs also showed elevated levels of expression of human VEGFR2, FGFR2, CXCR1, 2 and 4 receptors. Moreover, human CSCs growing in SCID mice stimulated murine stroma to produce elevated levels of angiogenic and growth factors.

**Conclusions/Significance:**

These findings suggest that chemotherapy can lead to propagation of CSCs and prevention of their differentiation. The high tumorigenic and metastatic potentials of CSCs are associated with efficient cytokine network production that may represent a target for increased efficacy of cancer therapy.

## Introduction

In recent years, substantial experimental evidence has been generated in support of the role of a small population of self-renewing cells that could sustain malignant growth [1], [2]. This population was termed cancer-initiating cells or cancer stem cells (CSCs) for their high capacity for self-renewal, multilineage differentiation, and superior levels of malignancy. CSCs have been identified and isolated in various malignancies including breast, brain, prostate, pancreatic, lung, and colon cancer [3]–[11].

CSCs were identified utilizing flow-cytometry-based cell sorting and NOD/SCID mice xenografting. CSCs express tissue-specific cell surface markers: e.g., breast CSCs express CD44+/CD24^low^ [3], and brain, prostate, lung and pancreatic CSCs express CD133+ [2], [4], [7], [8], [10].

Flow-cytometry-based cell-sorting which enables the isolation of a “side population” (SP) with enriched cancer stem cell activity was described by Goodell et al [12]. SP cells are characterized by distinct low Hoechst 33342 dye staining, attributed to the expression of ABCG2, an ATF-binding cassette (ABC) transporter [13]. SP cells also demonstrate a greater tumorigenic capacity than non-SP cells [13]–[15]. Flow-cytometry-based methods of sorting CSCs, using specific tissue CSC markers as well as the formation of spherical clusters of self-replicating cells [16]–[18], permit the isolation of a cell population enriched in early progenitor/stem cells.

Due to their high drug resistance and tumorigenicity, CSCs are thought to be responsible for tumor regeneration after chemotherapy [19], [20], although direct confirmation of this is still forthcoming. We therefore hypothesize that CSCs can be enriched and subsequently isolated from tumor cell populations following drug treatment.

In the present study drug surviving cells (DSCs) were isolated from human cancer cell lines treated with cisplatin, doxorubicin, or etoposide. Isolated DSCs exhibited high clonogenic capacities, enrichment with SP cells, expression of CSC cell surface and embryonic stem cell markers, a capacity for self-renewal, the generation of differentiated progeny, and high tumorigenic potential following SCID mice transplantation. We concluded that these DSCs were CSCs.

It has also been suggested that CSCs have high metastatic potential [21]. Recently, the relationship between pancreatic CSCs and tumor metastasis was demonstrated [8]. We demonstrated that drug isolated lung CSCs have high metastatic potential.

It continues to be unclear what properties of CSCs confer increased tumorigenicity and metastatic potential. We hypothesized that the tumorigenic and metastatic abilities of CSCs were based on their marked ability to produce growth and angiogenic factors, which stimulate tumor cell proliferation as well as promote formation of the tumor vascular system in order to provide oxygen and nutrients for local tumor growth or distant growth after dissemination of tumor cells into different anatomical locations. Thus, the highly efficient production of growth and angiogenic factors is a fundamental property of tumor-initiating cells. VEGF is a potent angiogenic factor [22], while growth factors such as bFGF, EGF, and HGF can stimulate proliferation of not only tumor cells but also endothelial cells and thus manifest proangiogenic and antiapoptotic effects [23]. Some data indicate that chemokines, such as IL-8 (CXCL8), MCP-1 (CCL2), and RANTES (CCL5), not only stimulate migration, but also proliferation of tumor and stromal cells, including endothelial cells [24]. Recently it was shown that IL-8 exhibits strong angiogenic activity via transactivation of VEGF receptor 2 (VEGFR2) [25]. Thus, different types of tumor producing factors (cytokines, chemokines, angiogenic and growth factors) have overlapping functions in promoting tumor growth. Numerous experimental and clinical data indicate that neutralization of growth or angiogenic factors, or blocking their receptor signaling, could inhibit tumor growth, confirming the importance of these factors in tumor cell proliferation [26]. Thus, production of growth and angiogenic factors by CSCs appears crucial for their tumorigenic and metastatic potentials. However, this CSC cytokine and growth/angiogenic factor network had not been previously investigated.

Therefore, in the present study, we performed a comprehensive analysis of various cytokines, chemokines, and growth factors produced by parental H460 tumor cells and isolated CSCs using multiplex xMAP technology (Luminex Corp., Austin, TX), which allows simultaneous analysis of numerous soluble factors. This analysis was performed *in vitro* on cultured cells and *in vivo* utilizing the human tumor xenografted model in SCID mice. Human tumors growing in SCID mice consist of human tumor cells and murine stroma. Sonicated extracts of the xenografted human tumors contained cytokines produced by human tumor cells as well as by murine stromal cells. Concentrations of each type of cytokines can be analyzed using multiplex kits developed specifically for the detection of human or murine cytokines. This could provide information about the cytokine network produced by CSCs and their ability to stimulate stroma formation.

Our studies demonstrate that drug surviving lung tumor cells have the characteristics of CSCs, and produce elevated levels of multiple cytokines, chemokines, growth and angiogenic factors and their receptors. These findings bring new insight to our understanding of the mechanisms responsible for high tumorigenic and metastatic potential of lung CSCs and their ability to survive chemotherapy.

## Materials and Methods

### Cell lines

Human cultured cancer cell lines, OVCAR-3 (ovarian), MCF-7 (breast), and H460 (lung), were obtained from the American Type Culture Collection (ATCC, Rockville, MD, USA). Cells were grown in culture media, as recommended by ATCC, supplemented with 20% FBS (Millipore Inc., Billerica, MA).

### Reagents

Cisplatin, doxorubicin, etoposide, and Hoechst 33342 were from Sigma-Aldrich (Sigma-Aldrich, St. Louis, MO). Fluorochrome-conjugated antibodies against human CD24, CD34, CD44, CD24, CD117, CD90, VLA-4, and VLA-5 were from Beckman Coulter (Fullerton, CA). Antibodies against FGFR2, VEGFR1, VEGFR2, and CXCR1, 2, 4 were from R&D Systems INC. (Minneapolis, MN). The antibody against VLA-6 was obtained from AbD Serotec (Raleigh, NY). Antibodies against CD 133 and cytokeratins 8/18 were from Abcam Inc. (Abcam, Cambridge, MA). Alexa Fluor®-488 conjugated mouse antihuman TRA-1-60, TRA-1-81, SSEA-1-4, and the antibody against human β-catenin were purchased from BD Biosciences Inc. (San Diego, CA). The antibody against phosphor-β-catenin was from Cell Signaling Technology Inc. (Beverly, MA). An embryonic stem (ES) marker sample kit, designed for detection of SSEA-1, 3, 4, TRA-1-60, TRA-1-81, and Oct-4, was obtained from Chemicon International (Tamecula, Ca). Alexa Fluor®-488 phalloidin and secondary Abs conjugated with Alexa 488, 546, and 680 were from Molecular Probes (Invitrogen, Carlsbad, CA).

### Clonogenic assays

Cells were plated at a density of 10–50 cells/cm^2^ in 100 mm^2^ Petri dishes or at a density of 0.5 cells/well in 96-well plates, and cultured for 14 days. For colony counting, cells were fixed and stained with Coomassie brilliant blue.

### Flow cytometry analysis

For side population (SP) analysis of cells we used standard protocol [12]. To inhibit ABCG2 transporter, 10 µM fumitremorgin C (Calbiochem/EMD Biosciences, Inc., San Diego, CA) was added 10 min before Hoechst addition. In some experiments, verapamil (50 µmol/L) was added with dye to confirm the SP (data not shown). Cells were analyzed using a MoFlo cytometer (Cytomation, Fort Collins, CO). Excitation of Hoechst dye was performed using a UV laser at 351 to 364 nm; the fluorescence was measured with a 515-nm side population filter (Hoechst blue) and a 608 EFLP optical filter (Hoechst red). Instrument gains were adjusted to set the main cell cohort, which comprises most of the cells containing one copy of DNA at the center of the plots. CD133+ cells were sorted from parental lung cancer H460 population using MoFlo cytometer and standard protocol for immunofluorescent staining.

### Cell staining procedure for Cellomics ArrayScan automated imaging

Cells were fluorescently stained as described [27]. Briefly, cells were grown in 96-well plates, washed with FACS buffer, incubated with antibodies against CD24, CD34, VLA-4, VLA-5, VLA-6, CD44, CD87, CD90, CD117, FGFR2, VEGFR1 and VEGFR2 conjugated with FITC, PE, or PC5, for 1 h, fixed in 2% PFA for 20 min, washed in PBS, and stained with Hoechst 33342. To test CD133, CXCR1, CXCR2 and CXCR4 expression, cells were incubated with respective primary antibodies and then with secondary antibodies conjugated with Alexa 488, 546, or 680 fluorochromes (Molecular Probes/Invitrogen) for 1 h. Cells were then stained with Hoechst 33342.

To detect intracellular proteins, cells were fixed, permeabilazed, and incubated with primary antibodies against embryonic stem cells markers, β-catenin, and cytokeratins 8/18 for 1 h and with secondary antibodies conjugated with Alexa 488, 546, or 680 fluorochromes (Molecular Probes/Invitrogen) for 1 h. For actin cytoskeleton staining, cells were incubated with Alexa Fluor 488 Phalloidin for 1 h. Cell nuclei were then stained with Hoechst 33342 at 2 µg/ml for 20 min to identify individual cells and to optimize focusing.

To stain tumor spheres, all manipulations were done under the microscopic control: tumor spheres were gently placed into individual wells of ultra low adherent 96 well plates, and incubation with primary and secondary antibodies was the same as described above for the adherent cultures.

All incubation and fixation procedures were performed at room temperature.

### Cellomics ArrayScan automated imaging

The Cellomics ArrayScan HCS Reader (Cellomics/ ThermoFisher, Pittsburgh, PA) was utilized to collect information on distribution of fluorescently labeled components in the stained cells. The ArrayScan HCS system scans multiple fields in individual wells, acquiring and analyzing each of the cell images according to defined algorithms. The scanner is equipped with emission and excitation filters (XF93, Omega Optical, Brattleboro, VT, USA) for selectively imaging fluorescent signals. Data were captured, extracted, and analyzed with ArrayScan II Data Acquisition and Data Viewer version 3.0 (Cellomics), Quattro Pro version 10.0.0 (Corel, Ottawa, Ontario, Canada), and MS Excel 2002 (Microsoft, Redmond, WA).

### Culture of lung cancer spheres

Suspension growth was assessed in methyl cellulose-based (MC-based) medium as described [16], [17]. Briefly, H460 cells and drug selected cells were resuspended in 0.8% MC-based serum free medium (Stem Cell Technologies, Vancouver, Canada) supplemented with 20 ng/mL EGF (BD Biosciences), bFGF, and 4 µg/mL insulin (Sigma) and plated at 500–10000 cells/mL in ultra low adherent 24–96 well plates (Corning, Corning, NY). EGF, bFGF (20 ng/mL), and insulin (4 µg/mL) were added every second day for two weeks. The medium was replaced or supplemented with fresh growth factors twice a week. In order to assess the self-renewing potential of the cells, spheres were collected by gentle centrifugation, dissociated into single cell suspensions, filtered and cultured under conditions described above.

### Differentiation

Cells dissociated from spheres (third generation) were plated at 1×10^4^ cells/mL on 96-well plates precoated with Collagen IV (BD Biosciences) in culture media supplemented with 10% FBS without growth factors and transferred into new plates when cultures reached confluence. To test the self-renewing potential of differentiated cells, cells were transferred into semisolid serum-free media supplemented with EGF, FGF, and insulin and their ability to form tumor spheres was evaluated as described above. To perform phenotypic characterization of cells from spheres and cells after differentiation, cells were seeded in 96-well plates (5×10^3^ cells/well) and stained with various antibodies as described above.

### Chemotherapy resistance studies

H460 parental cells, cells obtained from lung cancer spheres, and three weeks after CSCs differentiation cells were plated into 96-well plates precoated with Collagen IV (BD Biosciences) and cultured in RPMI 1640 media supplemented with 10% FBS. After 24 h doxorubicin and cisplatin were added at the final concentrations (0.016–025 µg/ml and 0.165–3.30 µg/ml, respectively). After 72 h of treatment, cells were stained with Hoechst 33342, and the number of cells per well were counted with Cellomics Array Scan VTI (Cellomics/ ThermoFisher, Pittsburgh, PA), as described [27].

### Migration and invasion assay

The migration and invasion activity of tumor cells in response to human recombinant IL-8 (100 ng/ml) was measured in BD BioCoat Matrigel Invasion Chambers (BD Biosciences, San Jose, CA) according to manufacturer protocol.

### Analysis of tumorigenic and metastatic properties of DSCs and H460 tumor cells

Experiments were carried out in accordance with guidelines provided by the Pittsburgh University Institutional Animal Care and Use Committee (IACUC) and the National Institute of Health Guide for the Care and Use of Laboratory Animals. SCID mice, 7−8 weeks old (Jackson Laboratories, Bar Harbor, ME), were maintained in the animal facility of the University of Pittsburgh Cancer Institute (UPCI).

To compare tumorigenic properties, H460 cells and DSCs were harvested and injected (in 200 µl of PBS) subcutaneously (s.c.) into SCID mice at concentrations of 5×10^3^–5×10^5^ cells per mouse (5 mice per group) without Matrigel. Tumors were measured twice a week. Mice were sacrificed when their tumors measured approximately 2 cm in diameter.

To analyze the ability of H460 cells and CSCs to form metastases, 5×10^4^ cells were inoculated intravenously (i.v.) into the tail vein of SCID mice. The SCID mice were T, B, and NKT cell-deficient but had active NK cells that could eliminate human tumor cells circulating in the blood stream. To deplete NK cells, SCID mice were inoculated intraperitoneally (i.p.) with 0.2 ml of anti-asialo GM1 IgG (diluted 1:20) 24 h before i.v. inoculation of tumor cells [28]. After 60 days, mice were sacrificed; lungs, livers, and kidneys were removed and fixed in the Bouin's solution; and metastatic nodules were counted under a dissecting microscope.

### Preparation of tumor extracts

Tumors grown s.c. in SCID mice were removed and snap-frozen in liquid nitrogen. Frozen tumor tissues were cut, sonicated for 20 s, and centrifuged at 15,000 g for 10 min to remove cell debris. Tumor extracts were stored at −80 °C.

### Multiplex analysis of cytokines

Analysis of human cytokines and growth factors in cell culture medium and in sonicated tumor lysates was performed using multiplexing xMAP technology (Luminex Corp., Austin, TX). Multiplex kits for detection of 49 human cytokines: IL-1α, IL-1β, IL-2, IL-4, IL-5, IL-6, IL-7, IL-8, IL-10, IL-12p40, IL-13, IL-15, IL-17, GM-CSF, IFN-α, TNFα, MCP-1, MCP-2, MCP-3, IP-10, MIP-1α, MIP-1β, RANTES, VEGF, bFGF, G-CSF, EOTAXIN, HGF, MIG, GROα, sIL-2R, sVCAM-1, CTACK, LIF, M-CSF, NGF, PDGF-BB SCF, SCGF-β, SDF-1α, TNF β, TRAIL IFN-γ, EGF, TNFRI, TNFRII, DR5, IL-1Rα, and sIL-6R were purchased from BIO-RAD Laboratories (Hercules, CA). Multiplex kit for detection of sFas, sFasL, TGFα, Fractalkine, sCD40L, TRAP, CS154, MIF, sVCAM-1, sICAM-1, MPO, Adiponectin, MMP-9, and tPAI-1 were purchased from Linco/Millipore (St. Louise, MO). The kit for detection of MMP-2 and MMP-3 was purchased from R&D Research (Minneapolis, MN). The multiplex kit for detection cancer antigens CEA, CA-125, CA 19-9, CA 15-3, CA72-4, AFP as well as mesothelin, IGFBP-1, kallikrein 10, EGFR, ErbB2, and Cyfra 21-1 were custom-made at the UPCI Luminex Core Facility (www.upci.upmc.edu/facilities/luminex). Mouse cytokines were analyzed using 19-plex kit for IL-1α, IL-1β, IL-2, IL-4, IL-5, IL-6, IL-10, IL-13, IL-17, IFN-γ, MIG, GM-CSF, MIP-1α, IL-12p40/p70, KC, TNFα, MCP-1, VEGF, and bFGF (Invitrogen/Biosource). Analyses of tumor supernatants and sonicated tumor extracts were performed in 96-well micro plate format according to manufacturers' protocols as previously described [29]. Parts of tumor extracts were used for protein analysis. Data were presented as mean±SD pg/mg of protein.

### Statistical analysis

All experiments were repeated at least three times. Comparisons between values were performed using a two-tailed Student's t-test. For the comparison of multiple groups, a one- or two-way ANOVA test was applied. Statistical analysis of the metastatic nodules was performed using Mann-Whithey test. For all statistical analyses, the level of significance was set at a probability of <0.05.

## Results

### Isolation of CSCs based on their resistance to chemotherapeutic drugs

**Figure 1:**
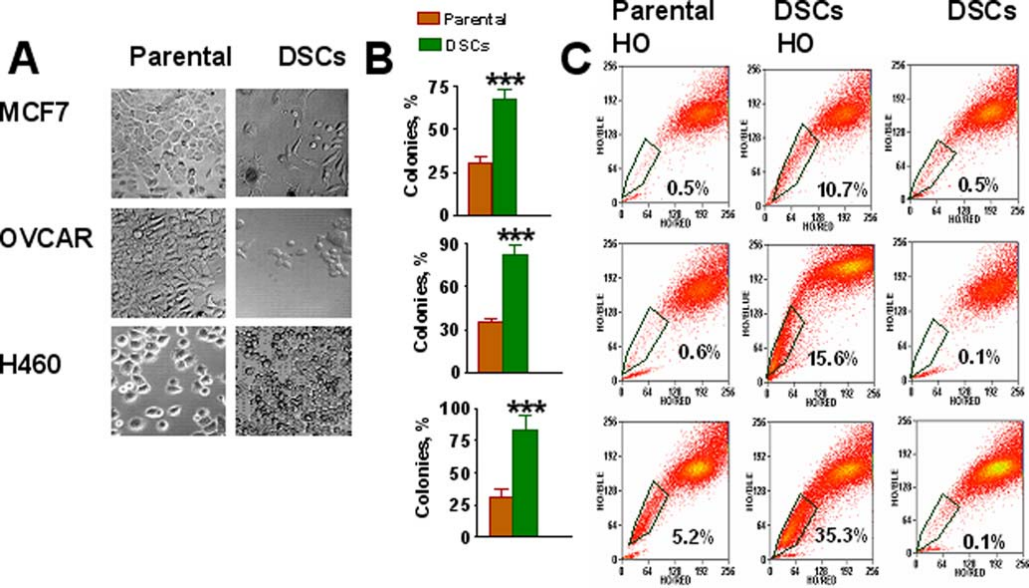
Selection of DSCs populations from human carcinoma cell lines. *A, Morphology of parental MCF7, OVCAR3 and H460 cells and drug survived cells (DSCs).* MCF-7 and H460 cells were treated with doxorubicin (0.125 µg/ml); OVCAR-3 cells were treated with cisplatin (3.3 µg/ml). After 48 h drugs were removed and drug-surviving cells (DSCs) were cultured for 3–4 weeks. *B, Increased colony formation by DSCs isolated from parental MCF7 (breast), H460 (lung) and OVCAR-3 (ovarian) cancer cell lines.* Cells were seeded 0.5 cell/per well in 96-well plates with culture media supplemented with 10% of FBS and cells were grown for two week. The percentage of colony formation was calculated. ***-P<0.001. *C, Analysis of side population (SP) in DSCs and parental MCF7, OVCAR-3 and H460 cell lines*. Tumor cells were stained with 5 µg/ml Hoechst33342 (HO). Some cells were pretreated with 10 µM fumitremorgin C (FTC) for 10 min prior to Hoechst addition (HO+FTC). Cells were resuspended in RPMI with 20% FBS and 2 µg/ml propidium iodide and sorted using MoFlo cytometer. Data for viable cells were analyzed for parametric correlations and annotated using FCS Express.

Ovarian OVCAR-3, breast MCF-7, and non-small cell lung cancer (NSCLC) H460 cells were treated with cisplatin (1–5 µM), etoposide (1–5 µM), or doxorubicin (0.067–0.125 µg/ml) for 3 days. A vast majority of the cells died. Over the next 7 days some surviving cells resembled senescent cells with enlarged and flattened morphology [30]. These “senescent” cells grew larger in size and died during weeks 2–4. During the first week after drug treatment, small, round, or spindle-shaped cells with lower adherence were detected, and their growing colonies gradually replaced the “senescent” cells in drug-treated cancer cell populations (Figure 1A). We assumed that drug surviving small cells were CSCs. To verify this, the expanded drug surviving cells (DSCs) were analyzed for their clonogenic capacity, SP phenotype, CSC markers, self-renewal capacity, ability to differentiate, and tumorigenic and metastatic potential.

### Clonogenicity of DSCs

The clonogenic capacity of parental H460, OVCAR3, and MCF7 cells and DSC populations was tested as described in Materials and Methods. Less than 40% of parental cells were able to form clones, whereas the clone-forming capacity of DSCs was more than twofold higher (Figure 1B).

### Analysis of SP phenotype

Analysis of SP fractions revealed that tested parental cell lines differed in the proportion of SP fraction, ranging from 0.6% in OVCAR3, 0.5% in MCF7, to 5.2% in H460 cells (Figure 1C). SP cell fractions were substantially higher in DSC populations varying from 10.7% in MCF7, 15.6% in OVCAR3 to 35.3% in H460. A distinct low Hoechst 33342 staining of SP cells has been attributed to the expression of ABCG2, an ATF-binding cassette (ABC) transporter [13]. To evaluate ABCG2 transporter activity, fumitremorgin C (FTC), an ABCG2 specific inhibitor was used. The SP fraction of DSC cells decreased significantly in the presence of FTC (Figure 1C), thus confirming upregulation of ABCG2 transporter in DSCs.

### Analysis of CSCs and embryonic stem cell (ESC) markers

The Cellomics Array Scan HCS Reader (Cellomics/ ThermoFisher) was used for imaging and analysis of expression of CSCs and embryonic stem cell markers in DSCs. This approach is based on a combination of microscopy and flow cytometry methods in a 96-well format. The advantages of the approach include: 10 times less cells are needed than for flow cytometry analysis, multi-spectral fluorescence micro-imaging is automated, and images are stored, visualized and analyzed using powerful software applications.

The analysis revealed that the DSC population from MCF-7 cells were CD44 positive with low levels of CD24 expression (data not presented), which corresponds to the previously identified phenotype of breast CSCs [3]. The DSCs from the ovarian OVCAR-3 line expressed CD44+ and ES marker Oct-4 (data not shown).

**Figure 2:**
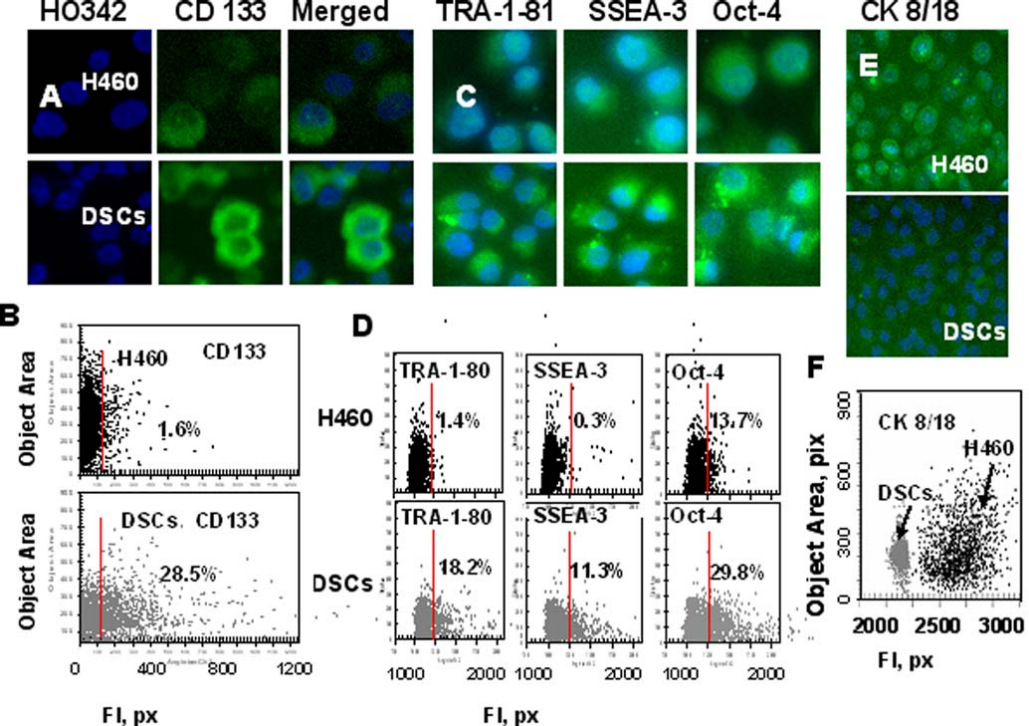
Analysis of CD133, embryonic stem cell (ESC) markers and cytokeratins 8/18 expression in H460 cells and DSCs. H460 cells and DSCs, growing in 96-well plates, were fixed and incubated with primary Abs against CD133, TRA-1-81, SSEA-3, Oct-4, or cytokeratins8/18 and then with secondary Abs. Cell nuclei were stained with Hoechst 33342. Cell images were acquired using the Cellomics ArrayScan HCS Reader (20X, 40X objectives) and analyzed using the Target Activation BioApplication Software Module. *A, Immunofluorescent images of tumor cells. B, Fluorescence intensity (pix) of CD133 plotted against object area.* Each point represents a single cell. Cells to the right of the red line are CD133+ (above IgG control staining). *C, Images of tumor cells immunofluorescently stained tumor cells for TRA-1-81, SSEA-3 and Oct-4* ES cell markers. D. Fluorescence intensity of TRA-1-81, SSEA-3 and Oct-4, plotted against object area. Each point represents a single cell. Cells to the right of the red line are positive (above IgG control staining). E,*Images of immunofluorescently stained tumor cells for cytokeratins8/18.* F, *Fluorescence intensity of cytokeratins8/18 in H460 cells (black dots) and DSCs (grey dots) plotted against object area*.

To date, human lung CSCs are poorly characterized [10], [15]. We therefore focused next on the characterization of CSC properties in DSCs from lung H460 tumor cell line. Analysis of CD34, CD24/CD44, CD87, and CD90 cell surface markers showed no differential expression between H460 parental and DSC populations (data not shown), whereas isolated human lung DSCs were enriched for the CD133+ population (Figures A, B). We next analyzed the expression of embryonic stem cell (ESC) markers, podocalyxin antigens, TRA-1-60, TRA-1-81, glycolipid antigens, the stage-specific antigens SSEA-3, 4, and transcription factor Oct-4, in H460 parental cells and isolated DSCs. Higher expression of TRA-1-81, SSEA-3, and Oct-4 was found in isolated DSCs as compared to parental H460 cells (Figure 2C, D), supporting our assumption that DSCs manifest markers associated with SCs.

### Low levels of differentiation marker cytokeratins (CK) expression in DSCs

**Figure 3:**
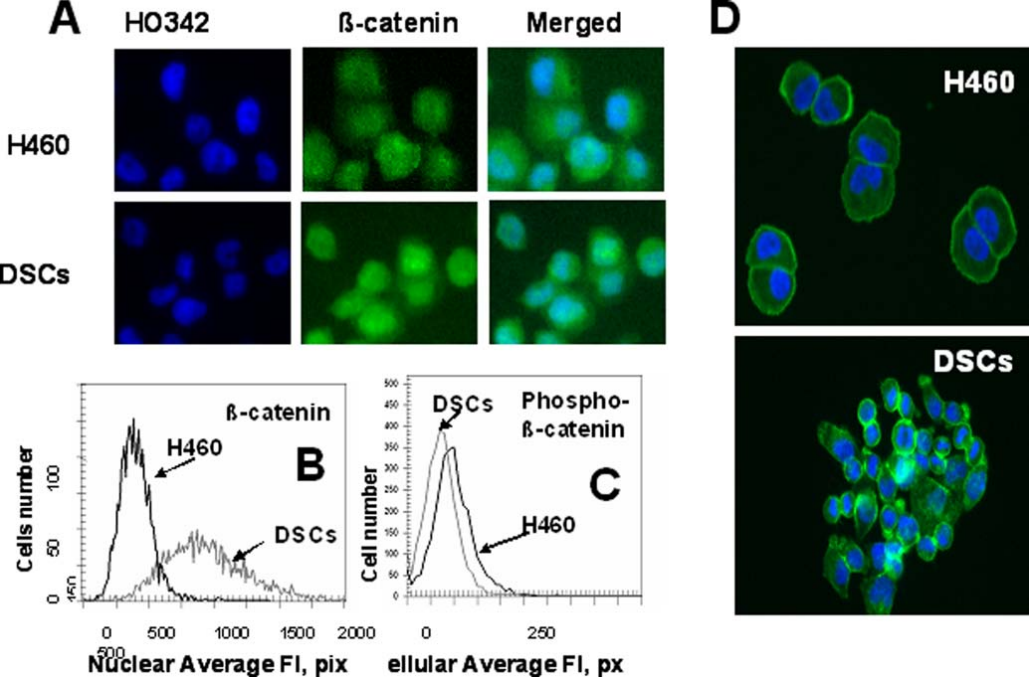
Analysis of β-catenin intracellular distribution in H460 cells and DSCs. Cells were fixed and incubated with Alexa Fluor® 488 phalloidin or with primary Abs against β-catenin and with secondary Alexa Fluor 488 conjugated Abs. Next cells were stained with Hoechst33342. Cell images were acquired using the Cellomics ArrayScan HCS Reader (20X objective) and analyzed using the Compartment Analysis BioApplication Software Module and the Target Activation BioApplication Software Module. *A, Images of H460 cells and DSCs immunofluorescently stained for β-catenin (A).* B, *An average fluorescence intensity of nuclear β-catenin in H460 (black line) and DSCs (grey line)*.C, *An average fluorescence intensity of cellular phosphor- β-catenin in H460 (black line) and DSCs (grey line). D, Cytoskeleton images of H460 cells and DSCs immunofluorescently stained for phalloidin and Hoechst33342.*

Lung cancer cells are known to express type I CK18 and type II CK8 cytokeratins, known differentiation markers in these cells [31]. We compared expression of CK8/18 in H460 cells and DSCs. In comparison to parental H460 cell line, DSCs expressed very low levels of CK8/CK18 (Figure 3D, E), indicating a low differentiation status of the isolated DSCs.

### β-catenin expression

Wnt signaling proteins have been shown to play a role in controlling stem cell self-renewal. β-catenin is a key player in the Wnt pathway, transmitting Wnt signals to the nucleus and playing a crucial role in tumorigenesis [32]–[34]. Here we analyzed the intracellular distribution of β-catenin in H460 cells and DSCs. DSCs showed substantially higher levels of total and nuclear β-catenin than parental H460 cells (Figure 3A,B), whereas phosphorylated β-catenin was present at low level in DSCs as compared to parental H460 cells (Figure 3C). It is known that in differentiated cells where Wnt signaling is absent, the level of β-catenin is regulated by a multiprotein “destruction complex” which binds and phosphorylates β-catenin, thus targeting it for ubiquitination and proteolytic degradation [32]. In stem cells where Wnt ligands are presented, this “destruction complex” is inhibited, preventing β-catenin phosphorylation and degradation, leading to stabilization and nuclear translocation of β-catenin [32]–[34]. Thus, high levels of nuclear accumulation of β-catenin and low levels of phosphorylated β-catenin suggest the active Wnt signaling in DSCs.

### Analysis of cell migration and expression of VLA adhesion molecules

Our morphological analysis of DSCs and parental H460 cells revealed differences in cell shape and adhesion properties, suggesting potential differences in their cytoskeletal organization. To test this hypothesis, we used Alexa 488 phalloidin to visualize the F-actin. As shown in Figure 3D, parental H460 cells have a round shape and uniform distribution of the F-actin in cytoplasm, whereas some DSCs have lamellipodial extension and actin spikes at the leading edge of the cells. These results suggest that DSCs have a greater “migratory phenotype” than parental H460 cells. We investigated the migratory capacity of H460 cells and DSCs using an *in vitro* migration and invasion assay using the IL-8 as the chemoattractant. Whereas, only 13.7% of parental H460 cells invaded through the Matrigel, 87.5% of DSCs were invasive.

**Figure 4:**
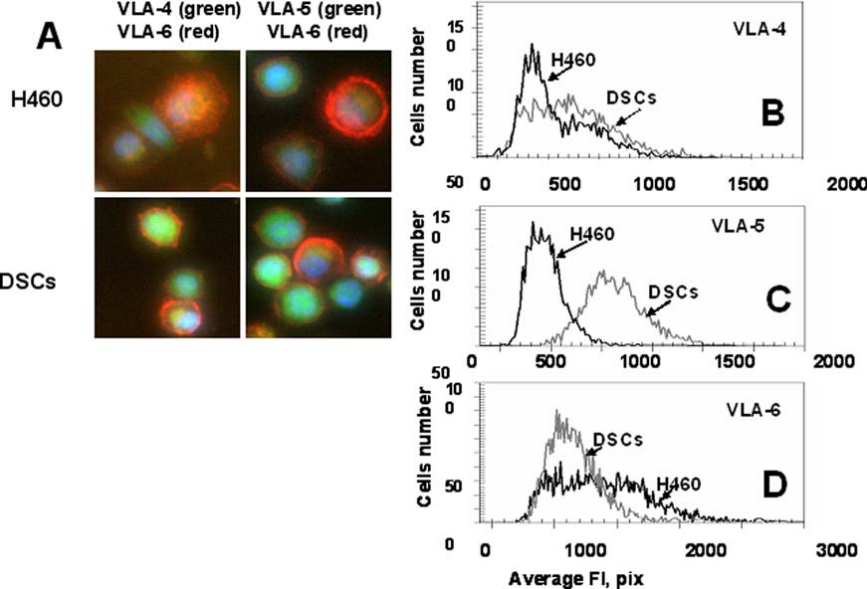
Expression of adhesion molecules, VLA-4(CD49d), VLA-5(CD49e), VLA-6(CD49f), by H460 cells and DSCs. Cells were incubated with Abs against VLA-4-FITC and VLA-6-PC5 or VLA-5-FITC and VLA-6-PC5. Cell images were acquired using the Cellomics ArrayScan HCS Reader (20X, 40X objectives) and analyzed using the Target Activation BioApplication Software Module. *A, Immunofluorescent images of VLA4/VLA6 (left) and VLA-5/VLA-6 (right) expression in H460 and DSCs cells (40X objective).* B-D, A*n average fluorescence intensity of VLA-4(B), VLA-5(C) and VLA-6(D) in H460 cells (black line) and DSCs (grey line).*

Adhesion molecules, including integrins, facilitate cell survival signaling and are involved in motility, intercellular adhesion, chemotaxis, and metastasis [35]. We compared the expression of integrins VLA-4, VLA-5, and VLA-6 in H460 cells and DSCs. We found that DSCs had significantly higher levels of VLA-5 and lower levels of VLA-6 as compared with parental cells, whereas VLA-4 levels were similar in both subpopulations (Figure 4D). Deprivation of tumor cell adhesion can trigger apoptosis. This form of apoptosis, induced as a result of the loss of cell's adhesion to a substrate, was termed anoikis. The low adhesion of DSCs may decrease their dependence on some surviving signals and lead to resistance to anoikis. Anoikis-resistant cells showed higher metastatic ability [36]. We observed that lung DSCs cultured under nonadherent conditions (low adherence plates) were resistant to anoikis, whereas all the H460 cells died under the same conditions.

### Chemotherapy selectively enriches for self-renewing lung cancer cells

**Figure 5:**
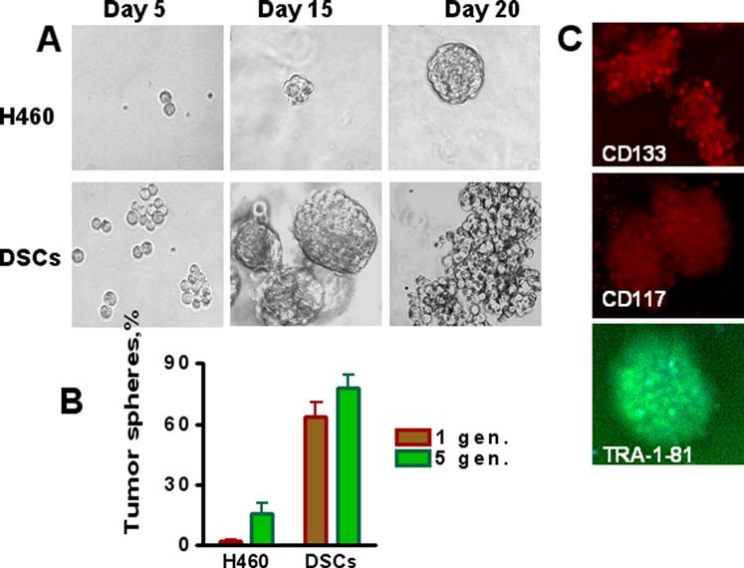
Chemotherapy selectively enriches for self-renewing lung cancer cells. Parental H460 cells and DSCs (1000 cell/ml) were plated onto ultra low adherent plates in MC-based serum free media supplemented with growth factors and cultivated as described in Material and Methods. Tumor spheres generated from single-cell suspension cultures of parental H460 cells and drug survived CSCs were counted after 3 weeks of culture (1 st generation), and then spheres were dissociated and replated as described in Material and Methods. *A, Lung tumor spheres generated from single-cell cultures of parental H460 cells and DSCs, imaged on indicated day of culture. B, Maintenance of enhanced ability to form tumor spheres during several generations* of DSCs transfer (for comparison only 1-st and 5-th generation's data are presented). C, *Immunofluorescent images of lung tumor spheres stained for CD133, CD117 and TRA-1-81 (10X objective).*

The ability to self-renewal and generate differentiated progeny are fundamental properties of CSCs. Tumor sphere generation is an *in vitro* assay of self-renewal potential [37]. Therefore, we assessed the self-renewal properties of DSCs by their ability to form tumor spheres when cultured in serum-free media and non-adherent conditions, as described in Materials and Methods. The vast majority of parental H460 cells died; however, a small proportion, 2.16%, of parental H460 cells survived and generated floating spherical colonies after 10–15 days in culture (Figure 5, A, B). In contrast, 63.71% of DSCs formed spheres. Moreover, the spheres, which developed from DSCs, grew faster and larger than spheres developed from untreated parental H460 cells. We next compared the self-renewal potential of cells derived from first generation spheres. Single cell suspensions were prepared from tumor spheres, transferred onto low adherence plates and cultured in serum free stem cells medium as described in Material and Methods. We found that spheres derived from DSCs produced a higher proportion of self-renewing (sphere forming) cells in comparison with sphere-derived H460 cells (Figure 5, B). Cells from spheres, regardless of whether they were derived from DSCs or parental H460 cells, expressed cancer stem cell markers CD133, CD117 (c-kit), and ESCs marker TRA 1-81 (Figure 5C).

### Analysis of DSCs ability to generate a differentiated progeny

**Figure 6:**
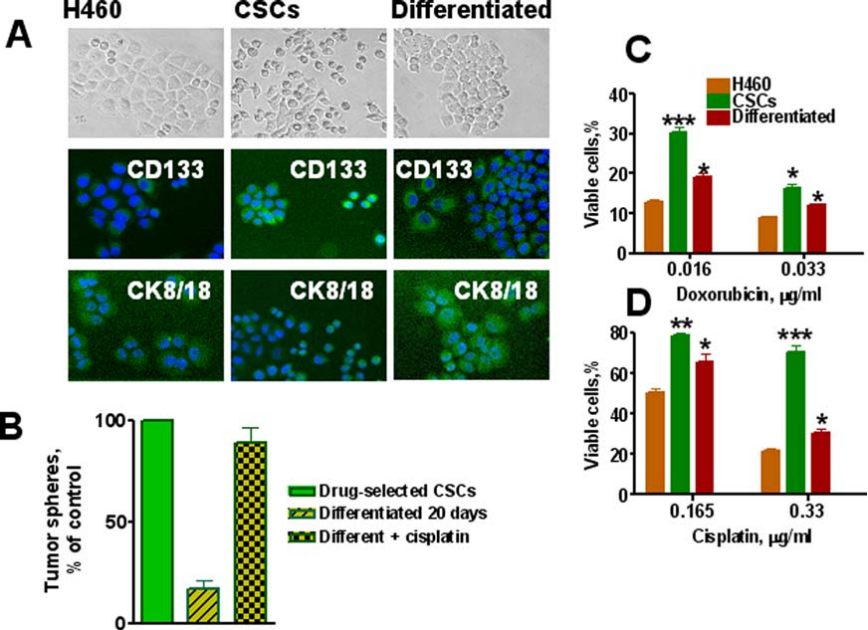
In vitro differentiation potential of lung cancer sphere cells and drug resistance of CSCs. *A, Loss of stem cell marker (CD133) and increase of differentiation markers (CK8/18) by lung CSCs differentiated progenitors.* Parental H460 cells and CSCs from tumor spheres were seeded in collagen coated well plates and cultured for 3 weeks in complete RPMI 1640 medium supplemented with 10% FBS. Upper row - cell images in phase –contrast microscopy; in the middle - cells immunofluorescently stained for CD133 and bottom row - cells immunofluorescently stained for CK 8/18. *B, Self-renewing ability of differentiated lung cancer cells treated with cisplatin*. Relative % of cells generated tumor spheres from single-cell suspension cultures of drug selected CSCs, cells differentiated during 3 weeks and Progenitors of CSCs differentiated for 3 weeks were treated with cisplatin (1 µM) for two days. Surviving cells were transferred into low adherent plates and cultured in semisolid serum free medium supplemented with growth factors. Numbers of formed tumor spheres were determined and presented as percent of control. Control is number of spheres formed by transfer of cells derived from control tumor spheres. Number of these spheres is accepted as 100 %. *C, Effect of cisplatin and doxorubicin on proliferation of parental H460 cells, CSCs and their differentiated cells*. H460, lung CSCs and differentiated cells were plated in 96-well plates precoated with Collagen at 1×10^4^ cells/well in complete RPMI 1640 medium with 10% FBS. After 24 h doxorubicin or cisplatin was added at the indicated concentrations. Cells were cultured for 72 h, fixed, stained with Hoechst 33342 (2 µg/mL), and counted using the Cellomics ArrayScan HCS Reader.

The differentiation potential of cells from third generation lung cancer spheres was evaluated. Cells dissociated from spheres were cultured in RPMI 1640 medium supplemented with 10 % FBS in plates precoated with Collagen IV to improve cell adhesion. After 3 weeks of culture under adherent conditions, the cells acquired the typical morphologic features of parental H460 cells with increased expression of the differentiation marker cytokeratins 8/18 and loss of expression of CSC marker CD133 (Figure 6A).

Next, we analyzed the self-renewal potential of differentiated cells. After 3 weeks of culture under adherent conditions, cells were transferred onto low-adherent plates and cultured in serum free stem cell medium. Tumor sphere formation was evaluated. Cells maintained under differentiating conditions for 3 weeks demonstrated a substantial reduction in their ability to form spheres, indicating a reduction in their self-renewal potential (Figure 6B).

### Effect of chemotherapy on parental H460 cells, self-renewing and differentiated lung cancer cells

We considered the question of whether chemotherapeutic drugs could select cells with self-renewing characteristics from already differentiated tumor cells. Cells that were differentiated for 3 weeks under adherent condition were treated with cisplatin (1 µM) for two days and high proportion of these tumor cells were killed by drug. The ability of cisplatin surviving cells to form tumor spheres in serum free medium supplemented with growth factors was analyzed. Untreated differentiated tumor cells displayed a low ability to form spheres, while cells that survived cisplatin treatment showed a high capacity for sphere formation and self-renewal (Fig. 6B). These data indicate that differentiated progeny of DSCs acquired drug sensitivity and were eliminated by subsequent re-exposure to drugs, whereas surviving cells represent a population with a high self-renewal capacity. To further confirm this conclusion, parental H460 cells, cells dissociated from tumor spheres, and cells differentiated in adherent conditions for 3 weeks were seeded into 96-well plates precoated with Collagen IV, and cultured for 3 days with different concentrations of doxorubicin or cisplatin. Surviving cells were counted using the Cellomics Array Scan. Parental H460 cells were highly sensitive to drugs, while cells from the tumor spheres were relatively drug-resistant (Figure 6C). Differentiated cells were more sensitive to drugs than sphere-derived cells, but slightly more resistant to drugs than parental H460 cells. These results demonstrate that differentiation of drug-resistant self-renewal cells is associated with increase their drug sensitivity. We repeated this cycle. The differentiated cells that survived drug treatment showed CSC characteristics and self-renewal. CSCs from the second round of selection were again able to develop differentiated progenitor cells that showed increased drug sensitivity as it was found during the first round of drug treatment (data not shown).

Taken together, all these data strongly indicate that DSCs express markers conventional for CSCs (CD133), ESC markers (TRA-1-81, SSEA-3, and Oct-4), low levels of differentiation markers CK8/18, and demonstrate a capacity for self-renewal and differentiation.

As shown above (Figure 2) parental H460 population contains 1.8% CD133+ cells. To test whether CD133+ cells from the parental H460 population share the markers of DSCs, we isolated CD133+ cells from parental untreated H460 cells using flow cytometry. Analysis of surface markers, CK8/18 expression, and the ability to grow in tumor spheres revealed that DSCs and CD133+ flow cytometry-sorted cells have the same phenotype (data not shown).

### DSCs have high tumorigenic potential

To compare the tumorigenic potential of drug-isolated CSCs in comparison with H460 cells, SCID mice were inoculated s.c. with 5×10^3^–5×10^5^ cells without Matrigel which provides artificial environment, stimulates production of various cytokine, and angiogenesis. As shown in Table 1, tumor growth was observed in all mice inoculated with 5×10^3^–5×10^5^ DSC cells, whereas no tumor growth was observed after inoculation with 5×10^3^ H460 cells. H460 cells grew in four out of five SCID mice inoculated with 5×10^4^ cells, and it required an injection of 5×10^5^ H460 tumor cells to develop tumors in 100% of SCID mice. Thus, DSCs demonstrated a substantially higher tumorigenic ability than H460 cells. Moreover, all tumors that developed from DSCs grew faster than those developed from parental cells as assessed by the time required for mice to bear tumors of 2000 mm^3^. All mice bearing DSC-derived tumors were sacrificed 2 wk earlier than animals inoculated with parental H460 cells. Tumor samples were frozen and used subsequently for cytokine analysis.

**Table 1:** Tumorigenic and metastatic properties of H460 cells and lung CSCs.

Subcutaneous tumors in SCID mice*		
No. of tumor cells inoculated	H460	CSCs
5×10^3^	0/5	5/5
5×10^4^	4/5	5/5
5×10^5^	5/5	5/5
Median No. of experimental pulmonary metastases (metastases in individual mouse)**		
No. of tumor cells inoculated	H460	CSCs
5×10^4^	0 (0,0,0,1,3)	58 (36, 47, 58, 173, 194)

* H460 cells and CSCs were injected s.c. into SCID mice at concentrations of 5×10^3^–5×10^5^ cells (in 200 µl PBS) per mouse. Mice were sacrificed when tumors reach 2 cm in diameter.

** H460 cells and CSCs were inoculated i.v. into the tail vein of SCID mice (5×10^4^ tumor cells/mouse). After 60 days mice were sacrificed, lungs were removed and fixed in the Bouin's solution, and metastatic nodules were counted under a dissecting microscope.

### DSCs show high metastatic capacity

We suggested that pulmonary metastasis formation following i.v. inoculation of tumor cells may be more indicative of the CSC nature of the DSCs lung tumor cells than subcutaneous tumorigenicity. It considered that metastatic nodules can originate from a single cell [38]. Therefore, the ability to form experimental metastases growing under orthotopic conditions in the lungs could be an ideal test for lung CSCs malignant potential. To compare metastatic ability, 5×10^4^ H460 cells and 5×10^4^ DSCs were inoculated i.v. into SCID mice. Sixty days after inoculation, metastatic nodules were found only in the lungs. It was also observed that parental H460 cells and DSCs differed dramatically in their capacity to develop lung metastases in SCID mice (Table 1). Whereas inoculated DSCs gave rise to multiple pulmonary metastases in all five animals (total of 508 metastases), inoculation with parental H460 cells resulted in the development of metastatic nodules in only two of five mice, with one and three metastatic nodules in each mouse. Thus, these results in combination with all in vitro experiments indicate that DSCs have all characteristics of CSCs. Hereafter DSCs will be termed CSCs.

### H460 cells and CSCs grown in SCID mice differ in cytokine production

The mechanisms responsible for the high tumorigenic and metastatic ability of CSCs remain unclear. We hypothesize that high tumorigenicity and metastastatic ability of CSCs are associated with their high ability to produce growth and angiogenic factors. These factors, through autocrine and paracrine mechanisms, support the proliferation of tumor cells and stimulate blood vessel formation that provide oxygen and nutrients essential for tumor growth. To test this, we analyzed various cytokines, chemokines, and angiogenic and growth factors in the lysates of H460- and CSC-derived tumors grown in SCID mice. Human tumors growing in SCID mice consist of human cells and murine stroma. This provides a unique opportunity to differentially analyze cytokines produced by human tumor cells and by murine stromal cells. For such analysis, we prepared sonicated lysates of tumors grown subcutaneously in SCID mice after inoculation of 5×10^5^ parental H460 cells or CSCs. Analysis of human cell-produced factors was performed using multiplex kits and Luminex technology for the detection of human proteins as described in Materials and Methods. The analysis revealed that human tumor cells growing *in vivo* produced a broad spectrum of cytokines and growth factors. Many factors were similarly produced by H460 and CSCs, such as IL-1β, IL-7, IL-10, IL-12p40, IL-15, MCP-2, RANTES, EOTAXIN, MIP-1β, IP-10, GROα, Fractalkine, sFAS, M-CSF, IL-1Rα, IL-2R, sIL-6R, and ErbB2. Nineteen different growth factors, cytokines, and chemokines were found to be substantially higher in the lysates of CSCs than in lysates of H460 tumors (Table 2). The levels of growth and proangiogenic factors VEGF, bFGF, IL-8, IL-6, HGF, PDGF-BB, G-CSF and IGFBP-1 were 2–3 folds higher in CSC tumors than in H460-derived tumors (Table 2).

**Table 2:** Multiplex analysis of cytokines and growth factors in the lysates of xenografted parental H460 and CSC-derived tumors.

Tumor Producing Factors		Mean±SE pg/mg of protein		
Cytokines		H460-derived tumor	CSCs-derived tumor	P value
1	**IGFBP-1**	18,853±1,583	62,090±6,210	<0.001
2	**VEGF**	3,218±516	8,249±980	<0.001
3	**IL-8**	6,295±905	10,360±700	<0.05
4	**IL-6**	1,808±184	3,599±479	<0.05
5	**bFGF**	941±84	3,055±657	<0.01
6	**HGF**	183±24	413±31	<0.001
7	**PDGF-BB**	8±1	24±6	<0.05
8	**SCGF-β**	1015±149	16,599±4,802	<0.001
9	**SDF-1α**	197±38	895±85	<0.05
10	**SCF**	61±4	80±1	<0.05
11	**G-CSF**	15±1	344±22	<0.001
12	**GM-CSF**	13±2	28±4	<0.01
13	**IFNα2**	94±13	203±27	<0.05
14	**MIP-1α**	18±1	38±5	<0.01
15	**MCP-1**	6±0.5	15±2	<0.01
16	**MIG**	8±0.8	16±1	<0.05
17	**PAI-1**	459±25	1,546±142	<0.01
18	**TNFα**	48±9	94±9	<0.05
19	**TRAIL**	116±23	231±23	<0.05

Sonicated extracts were prepared from H460- and CSC-derived tumors growing in SCID mice (5 tumors per group) and concentrations of various tumor-producing cytokines, chemokines, angiogenic and growth factors were analyzed using multiplex kits. Only factors with significant differences in their concentrations (at least p<0.05) are included.

The most remarkable differences were in the levels of stem cell growth factor-β (SCGF-β) in CSC-derived tumor lysates as compared to H460-derived tumor lysates. In addition, increased levels of stroma-derived factor-1α (SDF-1α) and stem cell factor (SCF) were found in lysates of CSC-derived tumors (Table 2). CSCs also produced significantly higher levels of chemokines (MIP-1α, MCP-1, and MIG), as well as INFα, TRAIL, and TNF-α (Table 2).

Taken together, these data demonstrate that high tumorigenic and metastatic potentials of CSCs correlate with superior production of angiogenic and growth factors involved in cell proliferation and angiogenesis. Increased levels of SCGF-β, SDF-1α, and SCF in tumors from CSCs are indicative of their stem cell origin.

**Figure 7:**
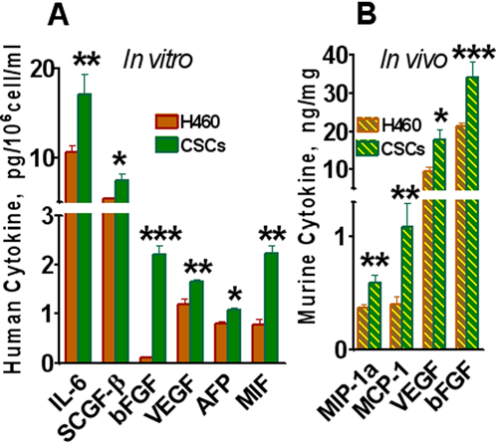
Multiplex analysis of cytokines. *A, In vitro cytokine production by CSCs and parental human tumor H460 cells.* Cells were cultivated in 96-well plates for 24 h in complete RPMI 1640 medium; samples of conditioned media were collected. Cells were fixed, stained with Hoechst 33342, and cell numbers were determined using image cytometry. Concentrations of human cytokines, chemokines, growth factors, MMPs, adhesion molecules and cancer antigens were analyzed using Luminex technology. Concentrations of cytokines pg/10^6^ cells/ml were calculated. Only factors with significant differences in their concentrations are presented. B, *Analysis of murine cytokines in extracts of xenografted parental H460 and CSCs-derived tumors.* SCID mice were inoculated s.c with 5×10^5^ of parental H460 or CSCs (5 mice per group). Samples of tumors, derived from parental H460 cells and CSCs, were sonicated, and concentrations of 19 murine cytokines in cellular extracts were measured using multiplexed cytokine assays as described in Materials and Methods. Only factors with significant differences in their concentrations (at least p<0.05) are included. Results are presented as pg or ng of cytokine per mg of total tumor protein.

H460 and CSCs cells cultured *in vitro* also showed differences in cytokine secretion. Lung CSCs produced twenty-fold more bFGF than H460 cells (Figure 7A). They also secreted higher levels of VEGF, IL-6, SCGF-β, and alpha-fetoprotein (AFP) than H460 cells (Figure 7A). In general, the spectrum of factors produced by these cells *in vivo* was much broader than those *in vitro*. This observation could be attributed to *in vivo* conditions being more conducive to the functional activity of tumor cells and their ability to produce various factors required for tumor growth.

### Analysis of MMPs and adhesion molecules in tumor samples

MMPs and adhesion molecules play a critical role in tumor invasion and metastasis [39], [40]. We analyzed the levels of three MMPs in the lysates of H460 and CSC tumors. Higher amounts of MMP-2 and MMP-3 were found in CSC-derived tumors than in H460 cell-derived tumors (Table 3), whereas no differences in expression of MMP-9 were observed. Higher levels of intercellular cell adhesion molecule-1 (ICAM-1) and vascular endothelial cell adhesion molecule-1 (VCAM-1) were detected in CSC-derived tumors. In addition, CSC-derived tumors contained higher levels of CYFRA 21-1 and mesothelin (Table 3), well-known lung tumor markers [41], [42].

**Table 3:** Multiplex analysis of adhesive molecules, MMPs and cancer antigens in the lysates of xenografted parental H460 and CSC-derived tumors.

Tumor Producing Factors		Mean±SE pg/mg of protein		
Receptors, adhesive and other molecules		H460-derived tumor	CSCs-derived tumor	P value
1	**DR-5**	589±43	783±40	<0.01
2	**TNF-R1**	162±23	546±70	<0.001
3	**VCAM-1**	2,190±112	3,922±604	<0.01
5	**ICAM-1**	137,207±7,385	185,007±12,176	<0.05
5	**Mesothelin**	54,617±3,956	106,625±17,695	<0.01
6	**Cyfra 21-1**	84,577±4,367	173,025±20.963	<0.01
	**MMPs**	**Mean±SE pg/mg of protein**		
7	**MMP-3**	undetectable	81±3	<0.001
8	**MMP-2**	8,129±250	9,767±560	<0.05
	**Cancer Antigens**	**Mean±SE pg/mg of protein**		
9	**CEA**	10,746±523	31,189±3,364	<0.05
10	**AFP**	14±2.7	25±2.8	<0.05
11	**CA 125**	28±3.6	43±3.7	<0.05
12	**CA 72-4**	5±1.9	310±48	<0.001

Sonicated extracts were prepared from H460- and CSC-derived tumors growing in SCID mice (5 tumors per group) and concentrations of various tumor-producing factors were analyzed using multiplex kits. Only factors with significant differences in their concentrations (at least p<0.05) are included.

### Analysis of cancer antigens

Many cancer-associated antigens are encoded by genes normally expressed in germ cells, trophoblasts, and embryonic cells [43]. We hypothesized that CSCs may express higher levels of embryonic antigens. To test this hypothesis, an analysis of serologically detectable cancer antigens (AFP, CEA, CA-125, CA 19-9, CA 15-3, and CA72-4) in the CSC- and H460-derived tumors was performed. Carcinoembryonic antigen (CEA) was the most prevalent cancer antigen in the tested tumor lysates regardless of origin; however, CSC-derived tumors contained three-fold higher CEA concentrations than parental H460-derived tumors (Table 3). The levels of AFP and CA 125 were almost two fold higher in CSC-derived tumors than in H460 tumors. The most dramatic difference was found in CA 72-4. The level of CA 72-4 detected in lysates of H460 cells was 5 pg/mg, whereas in CSCs tumors it reached 310 pg/mg of protein (Table 3). These data indicate that CSCs growing *in vivo* express higher levels of embryonic cancer antigens (CEA and AFP) as well as CA 125 and CA 72-4 when compared with parental cells.

### Mouse stroma-derived cytokines in human tumor xenografts

To measure cytokines produced by host stroma, multiplex kits for detection of 19 murine cytokines were used. Most factors were present in mouse tumor stroma at low or undetectable concentrations; however, levels of mouse proangiogenetic cytokines VEGF, bFGF, MCP-1, and MIP-1α in CSC-derived tumor samples were significantly higher than those in parental H460 extracts (Figure 7B). These results indicate that CSCs stimulate stroma formation more effectively than H460 cells. Of note, SCID mice lack T, B, and NKT cells, and thus stroma of xenografted human tumor is deficient in these and probably other inflammatory cells (macrophages, dendritic cells, neutrophils) that could contribute to a pool of stromal cytokines and chemokines.

Taken together, the comparative analysis of human factors produced *in vivo* and *in vitro* by CSCs and H460 cells show multiple differences in the range and amount of cytokines, thus highlighting the advantage of CSCs in proinflammatory niche formation and metastatic ability. Cytokines and growth factors exert their functions by binding to their respective receptors. Therefore, next we compared expression of growth and angiogenetic factors receptors in parental H460 cells and lung CSCs growing in adherent condition and in tumor spheres.

### Increased expression of growth factor and chemokine receptors by CSCs

Lung CSCs produced three-fold increased levels of VEGF (Table 2), a potent angiogenic factor which stimulates migration and proliferation of endothelial cells and formation of blood vessels by binding to its cognate receptors. Some evidence indicates that VEGF receptors (VEGFR1 and VEGFR2) are also expressed by tumor cells to facilitate pro-survival signaling that protects these cells from drug-induced apoptosis and stimulates their proliferation [44].

**Figure 8:**
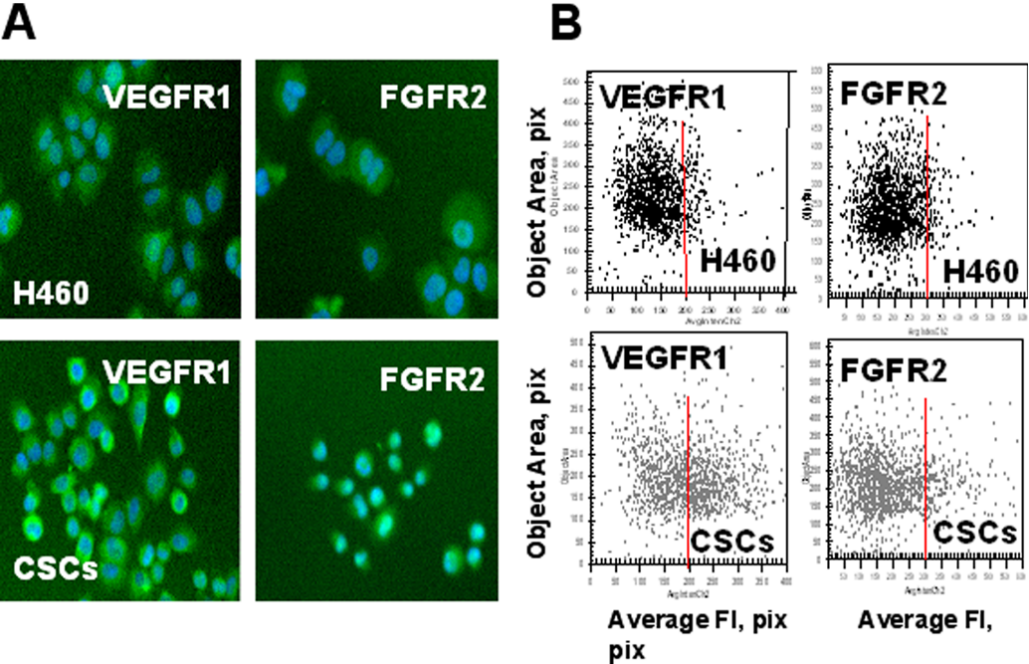
Increased expression of growth factor receptors (VEGFR1, FGFR2,) in lung CSCs. H460 cells and lung CSCs dissociated from spheres were plated into 96-well plates precoated with Collagen IV and cultured 8 h. Then adherent cells were incubated with FITC-conjugated Abs against FGFR2, VEGFR1 and VEGFR2 fixed and stained with Hoechst 33342. Images were acquired using the Cellomics ArrayScan HCS Reader (20X objective) and analyzed using the Target Activation BioApplication Software Module. *A, Immunofluorescent images of VEGFR1 and FGFR2 in H460 and CSCs cells (20X objective). B, Fluorescence intensity (pix) of VEGFR1 and FGFR2 plotted against object area.* Each point represents a single cell. In figures 8–10 red lines show the boundaries of the fluorescence intensity of H460 cells.

We used ArrayScan®VTI HCS Reader (Cellomics Inc) to identify VEGFR1 and VEGFR2 receptor expression in parental H460 cells and lung CSCs cultured under adherent conditions for 8 h. Both H460 parental tumor cells and CSCs expressed VEGFR1 (Figure 8A). However, lung CSCs showed higher levels of VEGFR1 expression than parental H460 cells (Figure 8B). The immunostaining of entire tumor spheres revealed high levels of VEGFR1 expression by CSCs (Figure 10C). VEGFR2 receptor was undetectable in analyzed cells.

FGF-b is an essential stemness supporting growth factor for both embryonic and cancer stem cells [45]. Additionally, it is a potent regulator of angiogenesis [46]. As we have shown above, lung CSCs produced an elevated level of bFGF both *in vivo* and *vitro* (Table 2, Figure 7A). We therefore expected that lung CSCs would express elevated levels of bFGF receptors as well. Indeed, expression of FGFR2 was elevated in lung CSCs growing in adherent conditions (Figure 8).

**Figure 9 pone-0003077-g009:**
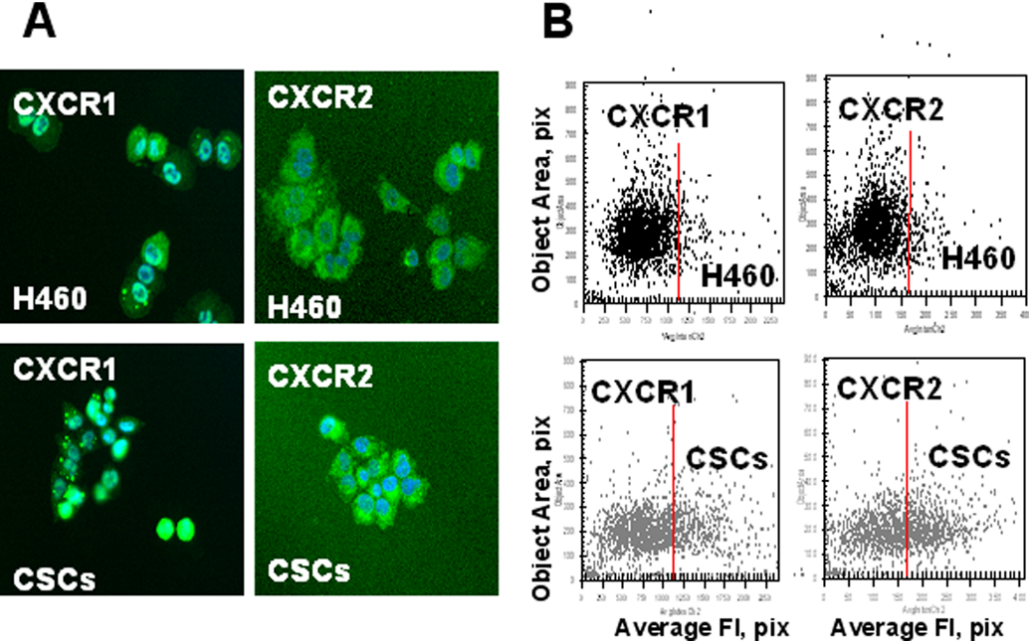
Increased expression of chemokine receptors (CXCR1, 2) in lung CSCs. *A, Immunofluorescent images of CXCR1 and CXCR2 in H460 and CS cells.* H460 cells and lung CSCs dissociated from spheres were plated into 96-well plates precoated with Collagen IV and cultured 8 h. Then adherent cells were incubated with antibodies against CXCR1 and CXCR2 and with secondary antibodies conjugated with Alexa Fluor® 488 and stained with Hoechst33342. Images were acquired using the Cellomics ArrayScan HCS Reader (20X objective) and analyzed using the Target Activation BioApplication Software Module. *B, Fluorescence intensity (pix) of CXCR1 and CXCR2 plotted against object area.*

**Figure 10:**
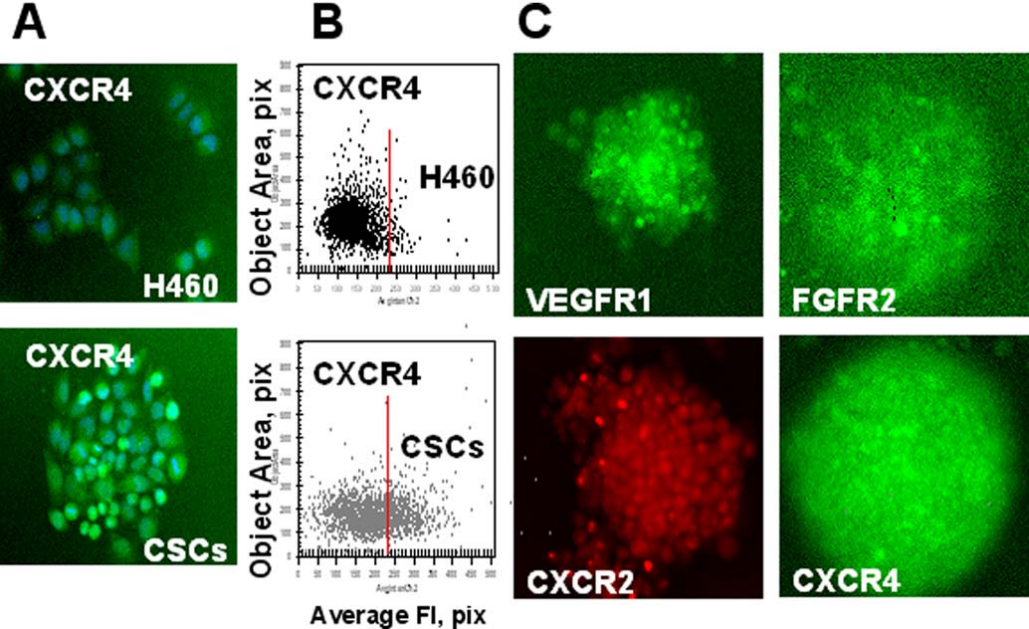
Expression of growth factor and chemokine receptors in lung CSCs. *A, B*, H460 cells and lung CSCs dissociated from spheres were plated into 96-well plates precoated with Collagen IV and cultured 8 h. Then adherent cells were immunofluorescently stained for CXCR4 (SDF-1 receptor); images were acquired using the Cellomics ArrayScan HCS Reader (20X objective) and analyzed using the Target Activation BioApplication Software Module. A. *Immunofluorescent images of CXCR4 in H460 and CSCs cells. B. Fluorescence intensity (pix) of CXCR4 is plotted against object area. C. Expression of growth factor and chemokine receptors in lung CSCs growing in tumor spheres.* Lung tumor spheres were immunofluorescently stained for VEGFR1; FGFR2, CXCR1 and CXCR4 receptors; images were acquired using the Cellomics ArrayScan HCS Reader (10X objective). Immunofluorescent images of lung tumor spheres stained for VEGFR1, FGFR2, CXCR1 and CXCR4 are presented.

IL-8 (CXCL8) is well known multifunctional prosurvival and proinflammatory chemokine involved in tumorigenesis, angiogenesis, and metastasis formation [47]. Lung CSCs exhibited higher levels of IL-8 *in vivo* (Table 2) and *in vitro* (Figure 7A). We also found that lung CSCs express high levels of IL-8 receptors, CXCR1, and CXCR2 (Figure 9, 10) as well as FGFR2 in tumor spheres Figure 10).

Stromal derived factor-1 (SDF-1, CXCL12) and its receptor, CXCR4, were significantly elevated in lung CSCs when compared with parental H460 cells (Table 2, Figure 10).

Our results clearly show that lung CSCs are characterized by higher production of growth and angiogenetic factors as well as higher expression of their receptors. These upregulations in cytokine network could serve as the basis for the enhanced tumorigenic and metastatic potentials of lung CSCs.

## Discussion

Our findings demonstrate that CSCs are resistant to chemotherapeutic drugs and can be selected from the parental population of human H460 lung tumor cells by treatment with drugs. Cells, which survived drug treatment, have all the properties of CSCs. Indeed, DSCs had high clonogenic efficiency, were enriched in SP phenotype, and expressed markers associated with CSCs, such as CD133. Expression of CD133 was found in CSCs of several different cancers, including brain, prostate, and pancreatic [2], [4], [7], [8]. Recently CD133+ lung CSCs were identified as well [10]. In our study we have shown that DSCs express putative embryonic markers Oct-4, SSEA-3, and TRA1-81 [6], [48], [49] and exhibit nuclear accumulation of β-catenin, which is believed to be a key event in stem cell activation [32]–[34], [50]. In parallel with expression of the phenotypic markers associated with CSCs, DSCs express low levels of epithelial differentiation markers (cytokeratins 8/18), further confirming the undifferentiated status of these cells.

Self-renewal and the ability to generate differentiated progenitors are considered fundamental properties of CSCs [1], [17]. We found that DSCs, in contrast to parental H460 cells, have a high ability to generate and maintain tumor spheres for numerous generations in selective culture conditions, indicating their high self-renewal potential. Furthermore, DCSs were able to differentiate and generate progenitors that acquired the differentiation markers cytokeratins 8/18 with parallel loss of CD133 and ability to form spheres. It is interesting that these differentiated tumor cells also acquired drug sensitivity, the vast majority of these cells were killed with cisplatin, and a small proportion of surviving cells maintained the ability to form spheres. Drugs did not avert CSC proliferation, but could prevent their differentiation, helping to preserve enrichment for CSCs in the population.

Finding that lung CSCs are resistant to different drugs (cisplatin, doxorubicin and etoposide) suggests the involvement of the complex mechanisms in their drug resistance that require further investigation. It is intriguing that changes during differentiation lead to the drug sensitivity of their differentiated progenitors.

The presence of CSCs in tumor cell lines propagated *in vitro* for many years put forth the idea that CSCs may be important for the maintenance of the tumor cell population not only *in vivo* but also *in vitro.* Elimination of drug sensitive differentiated tumor cells and enrichment of CSCs following drug treatment observed in our *in vitro* model of human lung cancer suggest that similar selection of drug resistant CSCs could be observed in clinical practice during chemotherapy. Recently it was reported that breast tumors from patients treated with chemotherapy contained higher proportion of CD44^+^, CD24^low^ cells with CSC properties than breast tumors from untreated patients [20].

High tumorigenic potential is a hallmark of CSCs. We found that DSCs, in comparison to parental H460 cells, have higher tumorigenic potential following s.c. inoculation into SCID mice. It has also been suggested that CSCs have high metastatic potential and can initiate the formation of distant metastases [21], although direct confirmation of this possibility still absent. It is widely held that metastatic tumors can develop from a single tumor cell. The metastatic process is highly selective and requires a tumor cell to survive during hematogenic spread and extravasation, followed by the development of supporting stroma and proliferation in distant tissues and organs [38]. We found that following i.v. inoculation of low amounts (5×10^4^) of tumor cells into SCID mice, DSCs formed numerous metastatic tumors in the lungs, whereas parental H460 cells failed to form any metastases in the majority of mice.

Thus, our *in vitro* and *in vivo* experiments demonstrate that DSCs possess all known characteristics of CSCs. What remains unknown; however, are the precise mechanisms by which CSCs are highly tumorigenic and metastatic. We found increased levels of adhesion molecules (VLA-5, ICAM-1, and VCAM-1) as well as MMP2 and MMP3 that could be contributing factors to the metastatic potential of CSCs. Recently it was shown that the CD133+ CXCR4+ pancreatic CSC population is the only cell population responsible for tumor metastasis in pancreatic cancer [8]. This finding appears to agree with our own in which lung CSCs isolated by drug treatment were CD133+ with increased levels of CXCR4 expression.

The high tumorigenic and metastatic properties of CSCs could be based on their increased ability to produce cytokines, chemokines, and angiogenic and growth factors, which, through autocrine and paracrine mechanisms, stimulate proliferation of tumor cells and migration and proliferation of stromal cells to form of a network of blood vessels that support tumor growth. We performed a comprehensive analysis of these factors in sonicated lysates of xenografted tumors derived from parental H460 cells and CSCs. We found that CSCs, in comparison with H460 tumor cells, produced up to two to threefold higher level of angiogenic and growth factors (VEGF, PDGF-BB, bFGF, IGFBP-β, and HGF). Moreover, lung CSCs showed higher levels of VEGFR1 and FGFR2 receptors. FGFb is a known fundamental growth factor, supporting the maintenance of embryonic stem cell and CSC populations [45]. Remarkably, CSC tumor lysates contained a very high level of SCGF-β and increased levels of SCF and SDF-1 – cytokines associated with the stem cell phenotype [51]–[53]. CSC-derived tumors have elevated levels of G-CSF and GM-CSF, which stimulate proliferation and differentiation of bone marrow cells and play a role in endothelial progenitor cell mobilization and proliferation via activation of STAT3 and STAT5 and upregulation of VEGF and its receptor, VEGFR2 [54].

CSC-derived tumors contain increased levels of proinflammatory cytokine IL-6 and chemokines IL-8 (CXCL8), MCP-1 (CCL2), and MIP-1α (CCL15), which are potent stimulators of angiogenesis and tumor cell proliferation and play an important role in tumor progression and metastasis in a variety of human cancers, including lung cancers [33]. In addition, we observed increased expression of both IL-8 receptors, CXCR1 and CXCR2, in lung CSCs. The chemokines and growth factors produced by the tumor by binding to the cognate receptors on tumor and stroma cells could provide proliferative and anti-apoptotic signals helping the tumor cells escape drug-mediated destruction. Indeed, we recently demonstrated that supernatants conditioned by tumor cells stimulate tumor cell proliferation and increase their resistance to chemotherapeutic drugs. Antibodies that neutralized tumor cell produced chemokines increased their sensitivity to drugs [55].

Human tumors growing in SCID mice consist of human cells and murine stroma. Multiplex analysis of murine cytokines and growth factors revealed an increased level of murine angiogenic factors VEGF, bFGF, as well as MIP-1α and MCP-1 in CSCs-derived tumors in comparison to parental H460 tumors growing in SCID mice, indicating that CSCs more efficiently stimulate murine proangiogenic factors and blood vessel formation to support the growth of xenografted human tumor.

Drug resistance of CSCs represents a serious obstacle for current chemotherapy-based treatment strategies, for which alternative approaches must be considered [56]. It is possible that CSCs might serve as a target for immunotherapy. Some cancer-associated antigens represent embryonic antigens that are predominantly expressed during embryonic development and completely or partially absent afterward. However, these antigens are often detected in malignantly transformed cells [43]. A substantial increase of embryonic antigens AFP and CEA was detected in CSC-derived tumors. Although the biological significance of these antigens remains unclear, it was recently reported that CEA inhibits anoikis by binding to TRAIL receptor DR-5 and preventing apoptotic signaling in tumor cells [57]. Our findings that that lung CSCs overexpressed CEA, DR5, and its ligand TRAIL provide strong support for this argument. In addition to AFP and CEA, CSC-derived tumors contained increased levels of CA 125 and CA 72-4, which are considered biomarkers of lung cancer [58] and provide additional target for immunotherapy.

Clinically, chemotherapy is typically administered in several cycles separated by three-week intervals in order to allow the body to restore hematopoietic and other normal cells damaged by drugs. However, some reports indicate that during this resting period tumor cells can aggressively repopulate the tumor and restore pre-treatment tumor size [59]. Based on our experimental data, it is plausible to conclude that chemotherapy increases the proportion of CSCs by elimination of drug sensitive differentiated tumor cells that could be restored during three weeks of rested period. Our data indicate that although differentiated tumor cells gain drug sensitivity, their populations keep higher proportion of CSCs and manifest higher drug resistance than original cell populations. With several cycles of chemotherapy the proportion of drug resistant CSCs or their early progenitors could further increase making the whole tumor even more resistant with each cycle of chemotherapy.

Further characterization of lung CSCs and the mechanisms of their drug resistance, as well as an investigation of their complex cytokine network, may provide key information on relevant pathways to be targeted to increase the therapeutic response.
